# Effects of surface defects on the mechanical properties of ZnO nanowires

**DOI:** 10.1038/s41598-017-09843-5

**Published:** 2017-08-25

**Authors:** Aditi Roy, James Mead, Shiliang Wang, Han Huang

**Affiliations:** 1 School of Mechanical and Mining Engineering, The University of Queensland, Queensland, QLD 4072 Australia; 2 School of Physics and Electronics, Central South University, Changsha, 410083 China

## Abstract

The elastic modulus of ZnO nanowires was measured using a resonance method based on laser Doppler effect and their fracture strains were determined via two-point bending with the aid of optical nanomanipulation. The elastic moduli of ZnO nanowires with diameters of 78 to 310 nm vary from 123 to 154 GPa, which are close to the bulk value of 140 GPa and independent of the diameters and surface defects. However, the fracture strains of the ZnO nanowires depend significantly on their diameters, increasing from 2.1% to 6.0% with the decrease in diameter from 316 to 114 nm. Post-mortem TEM analysis of the ends of the fractured nanowires revealed that fracture initiated at surface defects. The Weibull statistical analysis demonstrated that a greater defect depth led to a smaller fracture strain. The surface-defect dominated fracture should be an important consideration for the design and application of nanowire-based nanoelectromechanical systems.

## Introduction

ZnO nanowires (NW) are a promising material for the fabrication of future optoelectronic, electromechanical and electrochemical nano devices, due to their wide direct band gap (3.37 eV) and large excitation binding energy (60 meV)^1–3^. In addition, ZnO is a bio-safe and biocompatible material suitable for biomedical applications^4^. In order to improve reliability and optimize working conditions, it is of crucial importance to explore in-depth understanding of the mechanical properties of individual ZnO NWs, which are expected to be different from their bulk counterpart^5–8^. So far, considerable efforts have been made to study the mechanical properties of ZnO NWs. However, the reported results are largely scattered, some of which are contradictory to each other. For example, some studies have shown that the elastic modulus of ZnO NWs increases significantly with increasing diameter^9–13^, some other studies indicate that the modulus has a diameter-independent nominal value of ~ 140 GPa^14, 15^ and may even decrease with increasing diameter^16–18^. As the measurement is quite sensitive to the surface effects and structural defects of the testing NWs, as well as the testing strategies and environments^9, 12, 17, 19^, the accurate measurement and understanding of the mechanical properties of ZnO nanowires are still challenging^5–8^. For example, various experimental results indicate that the fracture strength of ZnO NWs depends strongly on their diameters, while two different mechanisms, surface-defect-dominated fracture^20^ and atomic-vacancy-dominated fracture^21^, have been proposed for explaining the size-dependent fracture strain/strength. Although both mechanisms are supported by different statistical analyses, no strong direct experimental evidences has been provided, due to significant difficulties associated with practical structural characterisation.

The mechanical properties of ZnO NWs were examined often using AFM-based three-point bending methods in an air atmosphere^14, 15^, or *in-situ* SEM/TEM methods where they are exposed to electron-beam irradiation^9–12, 19–22^. All these methods are able to detect the load-displacement relationship of the systems with ultrahigh resolution and thus are expected to achieve reliable results. However, for AFM-based three-point bending methods and *in-situ* SEM methods, it remains quite a challenge to obtain the structural information of the specific NW being tested and thus difficult to obtain a reliable relationship between mechanical properties and its structure^5–7^. *In-situ* TEM methods can directly examine the crystal structures of a NW, but possible effects from the electron-beam irradiation during the dynamic testing process are difficult to disregard^5–8^. As a result, some alternative strategies are indispensable for the mechanical characterization of NWs, such as nanoindentation-based methods^23, 24^, optical microscopy (OM) based methods^25–29^ and Raman spectroscopy based optic methods^30, 31^.

In our previous works, we developed a resonance method based on laser Doppler effect (LDE) and a two-point bending method based on OM nanomanipulation for measuring the elastic modulus and fracture strain of NWs, respectively^32–35^. In the present study, the mechanical measurement of individual NWs was performed under an optical microscope, followed by the examination of their morphology, structure and fracture surfaces using SEM and TEM. A significant influence of surface defects on the mechanical properties of ZnO NWs was confirmed by post-mortem TEM fracture analysis in conjunction with the Weibull statistical analysis. The methods adopted in this study effectively ruled out the effects of electron beam irradiation from *in-situ* SEM/TEM testing, demonstrating high potential to clarify the relationship between the measured mechanical properties and the structural characteristics of a NW.

## Experimental Details

### SLDV-based thermal resonance test

ZnO NWs were synthesized on a Si wafer by catalyst-free chemical vapour deposition^36^. The transfer of NWs was performed through OM nanomanipulation^34, 35, 37^ and resonance testing was performed using a Scanning Laser Doppler Vibrometry (SLDV, Polytec MSA-500; wavelength λ = 633 nm; power <1 mW; Objectives: Mitutoyo M Plan APO Plan 100×)^32, 33^. NWs were positioned at the frame edge of a SiN TEM grid to form NW cantilevers using a chemically etched W tip for resonance testing (The SiN TEM grid (product no. SN100-A05Q33) is commercially available, which is manufactured by TEMwindows.com (www.temwindows.com). The grid has a Si frame and a SiN film of 5 nm thick covering the grid holes. The surface roughness of the grid is 0.45 nm and the suspended 5 nm SiN film over the grid holes were peeled off via OM nanomanipulation, prior to resonance testing). Both NWs and substrate had atomically smooth surfaces, so the contact adhesion was sufficiently strong to securely clamp the NWs^32, 33^. Surface morphology, geometric dimensions and crystalline structure of the NW cantilevers were examined by SEM (JEOL JSM-7800F, operated at 10 kV) and TEM (FEI Tecnai F20, operated at 200 kV).

During the resonance test, the SiN TEM grid and positioned NW cantilevers were sealed in a vacuum chamber (air pressure ~10 Pa). The vibrational spectra of the NW cantilevers were obtained via SLDV through a sapphire window. According to the Euler–Bernoulli beam theory, the elastic modulus, *E*, of an ideally clamped NW with a uniform cross-section can be defined by^38^,

1 $$E=\frac{4\rho A{\pi }^{2}{L}^{4}{f}_{n}^{2}}{{\beta }_{n}^{4}I}\,,\,n\,=\,1,\,2,\,3,\ldots \,$$

where, *f*_*n*_ are the natural frequencies, *L* is the suspended length, *I* is the area moment of inertia, *A* is the cross sectional area and *ρ* is the density of the NW, while *β*_*n*_ (=1.875, 4.694, 7.855, … for *n* = 1, 2, 3, …) are the constants satisfying the transcendental equation, cos*β*_*n*_cosh*β*_*n*_ + 1 = 0. The dimensional parameters, *L* and *D* were all determined by SEM and TEM. The cross sectional area and area moment of inertia of the hexagonal nanowire were calculated by $A=3\sqrt{3}{D}^{2}/8$ and $I=5\sqrt{3}{D}^{4}/256$ respectively. The SLDV had the capability to capture multiple natural frequencies for each NW cantilever with high accuracy (typically less than 1% error). This provides an accurate criterion to examine whether or not the measured NW is well clamped and its geometry is uniform, by simply comparing the measured frequency ratios with the theoretical ratios^32, 33^,

2 $${f}_{1}:{f}_{2}:{f}_{3}:\cdots =1:6.27:17.55:\cdots .\,$$

### Two-point bending test based on OM nanomanipulation

The two-point bending tests of ZnO NWs were carried out on a SiN TEM grid, using the OM-based nanomanipulation^34, 35, 37^. The middle of a NW was first positioned on the suspending SiN film with its two ends attached on the TEM grid frame. One attached end of the NW was pushed by the W tip, along the surface of the grid frame (substrate), whilst the other remained at rest on the substrate due to strong adhesion with the supporting substrate. Once the NW was broken into two segments, the W tip was withdrawn from the substrate. The entire manipulation process was monitored using a digital video recorder. As the NW segments were placed on the suspending SiN film of a TEM grid, detailed morphology and fracture analyses could be handily examined by SEM (JEOL JSM-7800F, operated at 10 kV) and TEM (FEI Tecnai F20, operated at 200 kV). All optical tests were conducted in an ambient environment at temperature ~ 25 °C and relative humidity ~ 35%.

## Results and Discussion

**Figure 1:**
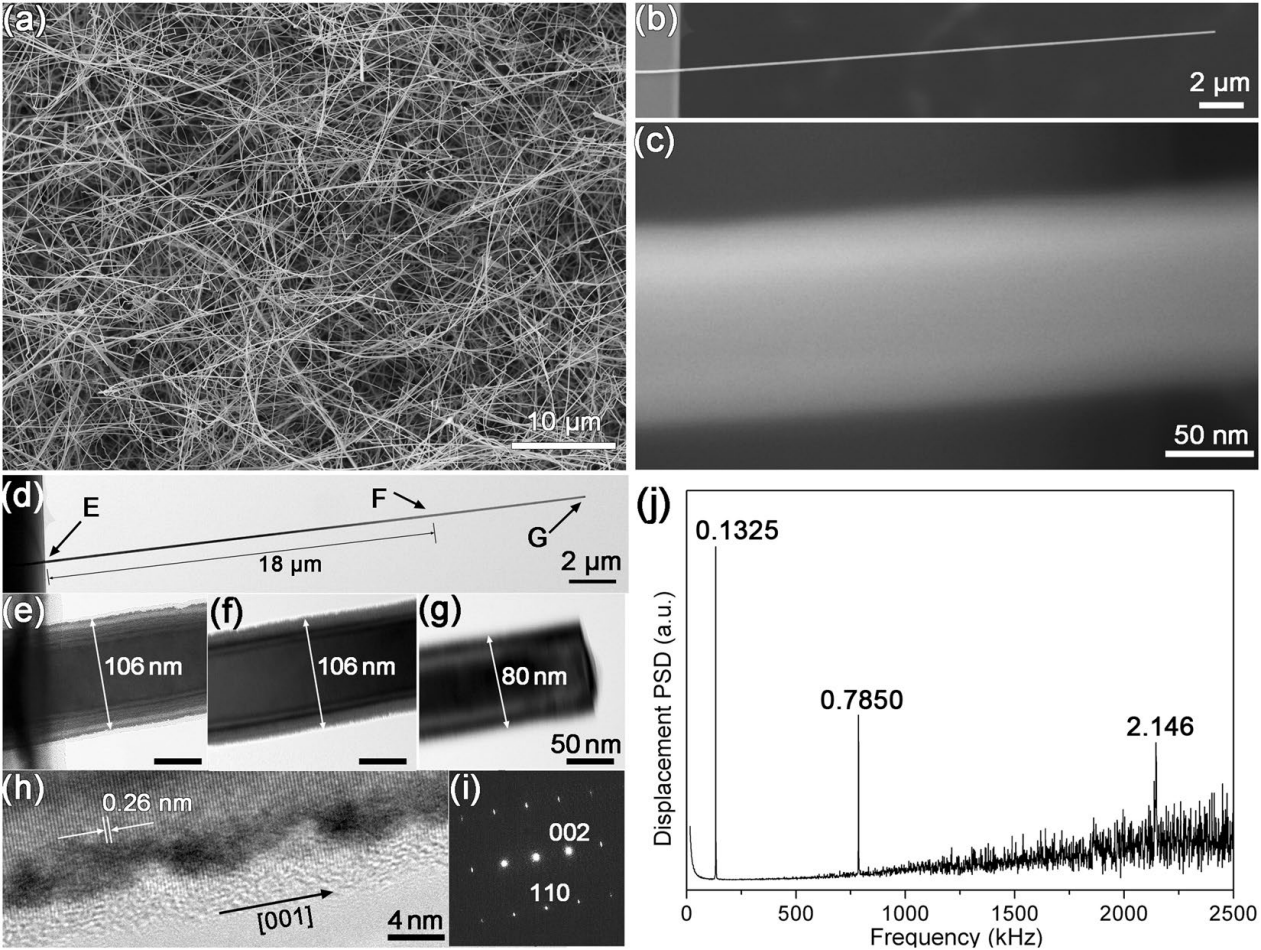
(**a**) Low-magnification SEM of the ZnO NWs fabricated on the Si substrate. (**b**) SEM image of a NW clamped at the frame edge of a SiN TEM grid, (**c**) High magnification SEM image of the NW with faceted surface. (**d**) Low Magnification TEM image of the same NW cantilever to (**b**). (**e–g**) High-magnification TEM images of the regions E, F and G of the NW shown by the arrows in (**d**). (**h**) HRTEM image and (**i**) the corresponding SAED pattern of the NW. (**j**) Vibrational spectrum of the NW cantilever.

Figure 1a shows a low-magnification SEM image of the ZnO NWs grown on a Si wafer using the catalyst-free CVD. Most NWs have a length of up to 100 µm. Figure 1b and d show the SEM and TEM images of the nanowires and Fig. 1j presents the vibration spectra of a typical NW. In Fig. 1b, it is shown that the NW’s a suspended length of 24.00 µm, clamped at the frame edge of a SiN grid by adhesion between the NW and SiN surface. The NW exhibits a faceted surface when observed at high-magnification, indicating that the NW has a hexagonal cross section (see Fig. 1c). The size and structure of the NW were determined directly by TEM, as shown in Figs 1d–i. The suspended length of the NW has a uniform diameter of 106 nm in the first 18 µm from its clamped end and then gradually tapers to 80 nm at its suspending tip (Fig. 1e–g). High-resolution TEM and selected area electron diffraction (SAED) analysis indicates that the NW has a hexagonal wurtzite structure (JCPDS: 36–1451, *a* = 0.324 nm, *c* = 0.519 nm) and grows along the [001] direction. TEM examination also indicates that the NW has a high-density of atomic-scale surface defects, as shown in Fig. 1h. Such surface defects were in fact observed in all NW cantilevers. Figure 1j shows the thermal vibrational spectra for the NW cantilever collected by SLDV. The first three natural frequencies, *f*_1_, *f*_2_ and *f*_3_, are 132.5, 785.0 and 2146 kHz, respectively. The measured ratio values, *f*_1_:*f*_2_:*f*_3_ = 1:5.92:16.20, are slightly discrepant from the theoretical values shown in Eq. (2). The effects of the tapered diameter on the ratio values of the natural frequencies can be simulated by a finite element model (FEM), using ANSYS software. All geometric parameters used in FEM (SOLID186 elements) were obtained from SEM and TEM measurements. The NW density of 5.6 g/cm^3^ Poisson’s ratio of 0.3 and elastic modulus of 140 GPa that are for the bulk ZnO were used^1^. FEM simulation indicated that the ratio values for the well-clamped NW, ${f}_{1}^{s}:{f}_{2}^{s}:{f}_{3}^{s}=1:6.02:\,16.23$, were comparable to the measured values. It is therefore confirmed that the small discrepancy between the measured ratio values and the theoretical values in Eq. (2) can be attributed to the tapered end of the NW and that any uncertainty resulted from this boundary conditions could be neglected. Precise modulus values of 146, 141 and 144 GPa were also obtained by matching the three natural frequencies in FEA.

**Figure 2:**
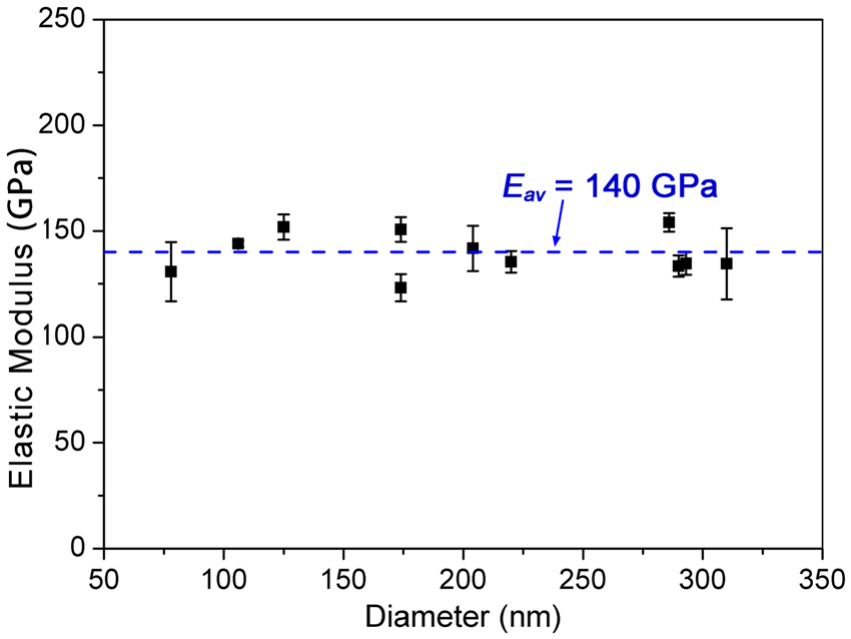
Elastic modulus of ZnO NWs plotted as a function of the wire diameter.

In this study, 11 ZnO NWs were tested, with diameters ranging from 78 to 310 nm, as shown in Fig. 2. The average elastic modulus being measured is 140 ± 10 GPa, indicating that the elastic moduli appears independent of the diameter and equals the value of 140 GPa for bulk ZnO^1^. Several factors should be responsible for this result. First, our ZnO NWs have relatively large diameters of above 78 nm, which are greater than the critical size of 30 nm for diameter-dependent modulus obtained from the molecular dynamics simulation^10^. Second, our tests were carried out in vacuum and power of the laser beam is quite low (small than 1 mW), which prevented the possible effects from humidity^17^ and averted the electron-beam induced effects (such as electron-beam induced damage and electron-beam induced C deposition)^5^. Third, our comprehensive TEM examination indicated that the ZnO nanowires being used in this study are free of planar defects and the size of surface defects is considerably smaller than the NW diameter. As the elastic modulus of nanowires is much less sensitive to surface defects^15^, in comparison to the vacancies and planar defects inside nanowires^19, 39, 40^, it is expected that surface defects would have insignificant effect on the elastic modulus of nanowires. The previous studies also indicated that atomic vacancies might substantially decrease the elastic modulus of ZnO nanowires^19, 41^. Our modulus results didn’t demonstrate such influence. This might be because the number of atomic vacancies in our ZnO nanowires was too small to influence the elastic modulus.

**Figure 3:**
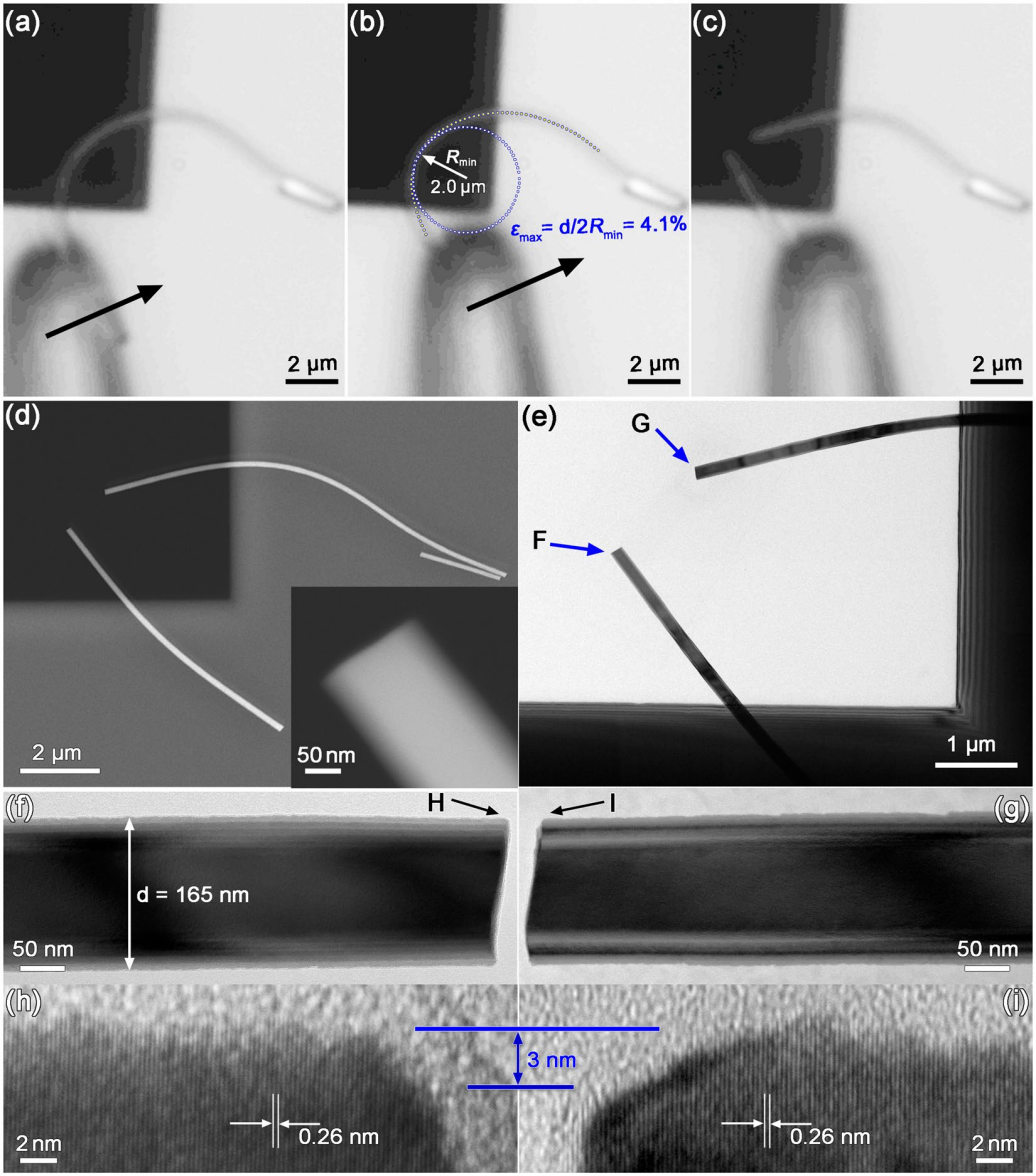
(**a–c**) Optical images extracted from the video slip, showing the bending test of a ZnO NW on a SiN TEM grid. The arrows in (**a**,**b**) show the direction of the push by a W tip and the dotted line circle is used to determine the curvature of the most bending shape of the NW right prior to fracture. (**d**,**g**) low-magnification SEM and TEM images of the ZnO NW after fracture. The inset in (**d**) shows the hexagonal morphology of the NW. (**f**,**g**) Enlarged TEM images of the two fracture segments. (**h**,**i**) High-resolution TEM images of the two fracture corners labelled by H and I in (**f**) and (**g**), respectively.

The fracture strengths of ZnO NWs were measured using two-point bending. Figures 3a–c show the optical images extracted from the video recorded during bending, demonstrating a ZnO NW being manipulated into a normal bent shape, the bent status just prior to fracture and the status after fracture, respectively. The arrows in Fig. 3a and b show the pushing direction of the W tip along the substrate surface. The profile of the bent NW was fitted using a polynomial and the radius of curvature, *R*, of the curve was then derived from the polynomial^42–45^. For the NW bent profile shown in Fig. 3b, the minimum radius of curvature, *R*_*min*_, is calculated to be 2.0 µm. The error associated with the polynomial fit in this procedure is estimated to be approximately 10%. The blue dotted line in Fig. 3b outlines a circle with a radius of *R*_*min*_, tangent to location on the NW where the radius of curvature is at the minimum. Fracture of the NW occurred at this location and the NW was broken into two segments, as shown in Fig. 3c. The morphology of the NW segments was characterised by SEM, as shown in Fig. 3d. Corresponding TEM analyses revealed that the NW had a single-crystalline structure with a uniform diameter, *d* = 165 nm, along the length (Fig. 3e). Figure 3f and g show the high-magnification TEM images of the fractured surfaces, F and G, as marked in Fig. 3e, where the NW has randomly distributed small surface defects. The surface defects have a typical depth of 1–2 nm, as can be clearly observed in the HRTEM images of the location of fracture initiation (Fig. 3h and i). As no evidences on plastic deformation exist on the fractured surface, the NW was broken in a brittle mode. Therefore, the strain,ε and strength, *σ*, could be calculated using the following equations, *ε* = *d*/2*R*_*min*_ and *σ* = *Eε*, respectively^34, 35^. For the NW shown in Fig. 3, we obtained: *ε* = (4.1 ± 0.4)% and *σ* = 5.8 ± 0.6 GPa by using *R*_*min*_ = 2.0 ± 0.2 µm, *d* = 165 nm and *E* = 140 GPa.

**Figure 4:**
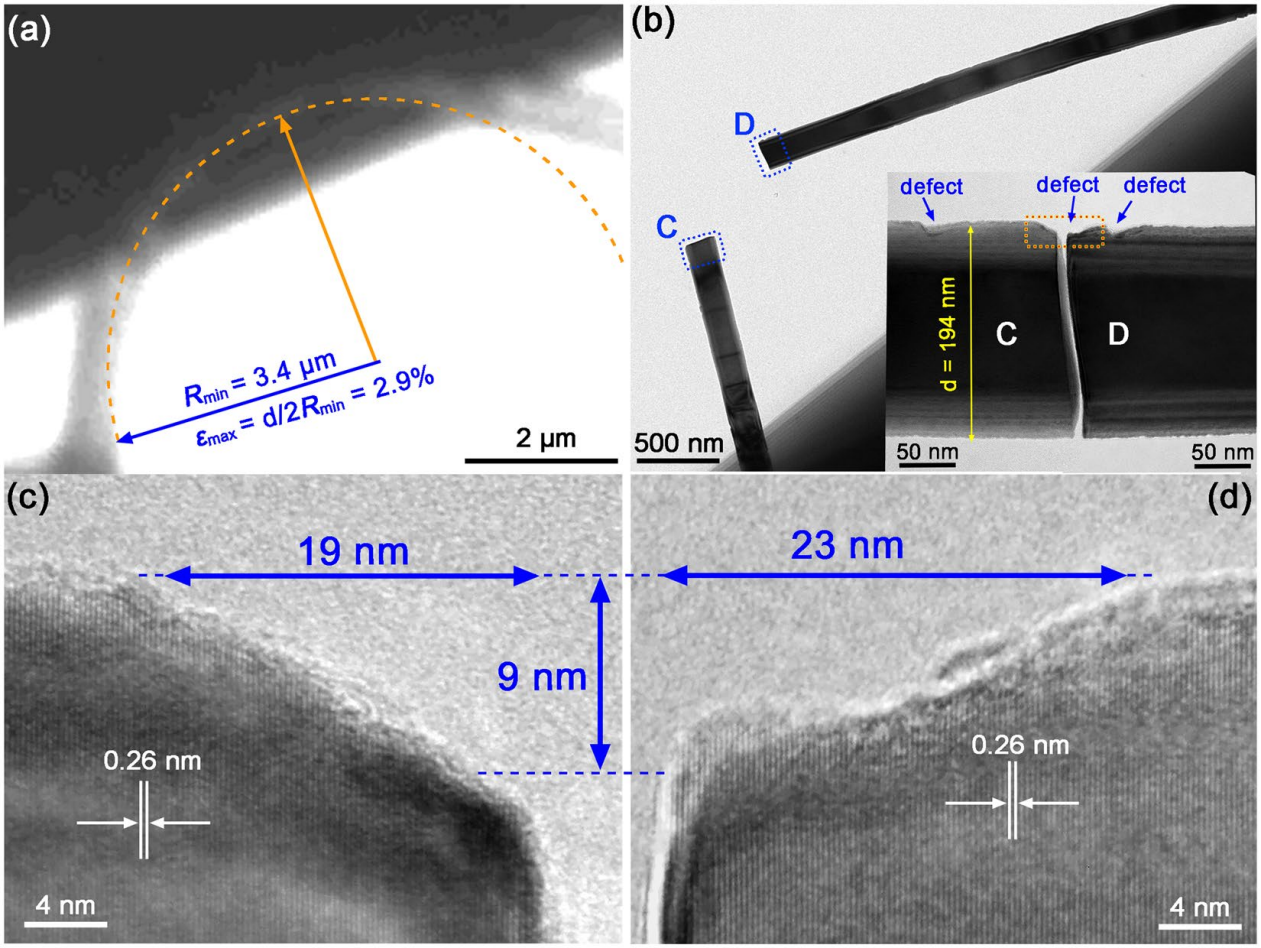
(**a**) Optical image of an elastically bent ZnO NW right prior to fracture. (**b**) TEM images of the two fracture ends, C and D, of the NW after fracture. The inset is the enlarged TEM images of the fracture ends C and D, showing that the fracture initiated at a relatively large surface defect. (**c**,**d**) HRTEM images of the fractured surfaces marked by the dotted line rectangle in (**b**), showing the defect at the initiation site of fracture.

**Figure 5:**
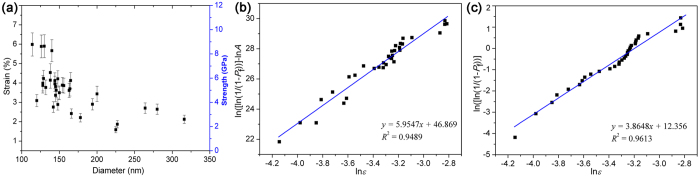
(**a**) Fracture strain and strength plotted as a function of the diameters of ZnO NWs. Strength distributions of the NWs using (**b**) three-parameter and (**c**) two-parameter Weibull statistical analysis.

Previous studies suggested that surface defects could be the main cause for the weakening of a NW’s strength, but it was unable to provide direct experimental evidences to verify this hypothesis^12, 14, 20, 21^. It is interesting to note in Fig. 3h and i that a surface defect of a depth of 3 nm can be identified at the corners of the fracture surfaces (F and G). This indicates that the fracture did initiate at the location of a surface defect. Our TEM analyses revealed that the NWs of a greater diameter had larger surface defects. In this work, the effect of surface defect size on the fracture strength of NWs was explored. Figure 4a shows an elastically bent NW right prior to fracture and Fig. 4b shows TEM images of the broken NW. A maximum strain of 2.9% was achieved for the NW of *R*_*min*_ = 3.4 µm and *d* = 194 nm. Similar to the breaking of the NW shown in Fig. 3, TEM analysis revealed the presence of a defect at the corners of the fractured surfaces, as shown in Fig. 4c and d. This suggests that fracture of the NW initiated at a relatively large defect of 9 nm deep and 42 nm wide. 33 NWs of the diameters ranged from 114 to 316 nm were tested and the fracture strains and strengths measured are plotted as a function of the NW’s diameter in Fig. 5a. It is clearly seen that the fracture strain and strength decreased with the increase in diameter. The maximum strain of 6.0% being achieved for the NW of 114 nm in diameter is comparable to the theoretical simulated values of 6.2%^46^, 6.5%^20^ and 7.5%^47^, as well as the previously experimental maximum values of 5.5%^14^, 6.2%^20^, 7%^22^, 7.2%^44^ and 7.7%^48^. It is believed that the discrepancy between the measured and theoretical values should be mainly attributed to the effect of surface defects.

To further understand the fracture behaviours of NWs, the measured fracture strains were analysed using the Weibull-type weakest-link probabilistic models. According to classical three-parameter Weibull statistics, for a NW with a mass of defects uniformly distributed throughout the surface, the probability of fracture (*P*_*f*_) under a strain is related to the surface area (*A*)^20, 49, 50^:

3 $${P}_{f}=1-\exp \,[-{({\varepsilon }_{f}/{\varepsilon }_{0})}^{m}A]\,$$

where *ε*_*f*_ is the failure strain and *ε*_0_ is the characteristic strain related to unit surface area and *m* is the Weibull modulus. The probability of failure *P*_*f*_ can be calculated as, ${P}_{f}({\varepsilon }_{i})=(i-0.5)/N$, where *N* is the total number of specimens tested and the observed strength values are ranked in ascending order as *ε*_1_, *ε*_2_, *ε*_3_, …, *ε*_*n*_. In our measurement, fracture initiates at the most outer layer of the NW, where the strain is normally greater than 1% (Fig. 5a). Therefore, the effective measured length, *L*_*eff*_, should be highly localized at the most curved part of the NW and thus can be roughly estimated as, *L*_*eff*_ = π*R*_*min*_. Therefore, the effective surface area of the NW, *A*_*eff*_, can be estimated by, *A*_*eff*_ = π*R*_*min*_ *d*. Based on this assumption, the classic Weibull statistics for the measured strain data with respect to the effective surface area is shown in Fig. 5b. The Weibull modulus, *m* and coefficient of correlation, *R* ^2^, are found to be 5.95 and 0.95, respectively. The relatively large values for *m* and *R* ^2^ indicate a strong correlation between the fracture strain and surface area and thus the number and size of surface defects^20, 49, 50^. Our results could be well fitted by the model in Eq. (3), suggesting that the number of atomic vacancies in the ZnO nanowires being tested should be very small^21^.

Analysis of the measured data was also undertaken using simple two-parameter Weibull statistics^21, 51–53^,

4 $${P}_{f}=1-\exp \,[-{({\varepsilon }_{f}/{\varepsilon }_{0})}^{m}],$$

where the probability of failure *P*_*f*_ is determined by a single critical defect or a very small number of critical defects and no longer related to the specimen sizes. Figure 5c shows the result fitted by the Eq. (4). The values of *m* = 3.85 and *R* ^2^ = 0.96 suggests that fracture should also be controlled by the critical defects present on a NW’s surface. The measured results are well fitted by both two-parameter and three-parameter Weibull statistics. This is quite different from the previously published results that show the probability of fracture could only be well fitted by either three-parameter or two-parameter Weibull statistics^21^. Variation in the fracture statistics may be explainable by considering the different mechanical characterisation strategies and NW samples used. First, the effective surface area, *A*_*eff*_, in the two-point bending test is highly localized at the most outer layer around the most bent region of the tested NW and thus significantly smaller than that in a tensile test, where *A*_*eff*_ is the whole surface area of a NW between the two clamped points. In this case, the fracture is expected to be controlled by a very small number of critical surface defects. Consequently, the fracture strain or strength sensitivity to a NW’s diameter over a relatively small diameter range is reduced and thus the two-parameter Weibull statistics are valid^22, 48, 53^. Second, the surface defect size present on the ZnO NWs investigated in this study increase with the increased diameter. If fracture is dominated by the defect size, then a decrease in fracture strain with increased NW diameter is quite reasonable. Therefore, the three-parameter Weibull statistics related to the surface defects are also valid.

## Conclusions

The elastic modulus and fracture strength of ZnO NWs were determined using the resonance and two-point bending methods, respectively. The moduli of NWs with diameters ranging from 78 to 310 nm were approximately 140 GPa ( ± 10 GPa), showing little effect of wire diameter. The fracture strength of the NWs increased from 2.9 to 8.1 GPa with a decrease in diameter from 316 to 114 nm. The fracture was found to initiate at the site of defect and the fracture strength being measured depends on the size of surface defects. TEM and Weibull statistical analyses also demonstrated that a larger NW had greater defects on the surface, thus leading to smaller fracture strength.
